# Versatile Porphyrin Arrangements for Photodynamic Therapy—A Review

**DOI:** 10.3390/nano14231879

**Published:** 2024-11-22

**Authors:** Arleta Glowacka-Sobotta, Beata Czarczynska-Goslinska, Daniel Ziental, Marcin Wysocki, Maciej Michalak, Emre Güzel, Lukasz Sobotta

**Affiliations:** 1 Chair and Department of Orthodontics and Temporomandibular Disorders, Poznan University of Medical Sciences, Bukowska 70, 60-812 Poznan, Poland; aglow@ump.edu.pl; 2 Chair and Department of Pharmaceutical Technology, Poznan University of Medical Sciences, Rokietnicka 3, 60-806 Poznan, Poland; bgoslinska@ump.edu.pl; 3 Chair and Department of Inorganic and Analytical Chemistry, Poznan University of Medical Sciences, Rokietnicka 3, 60-806 Poznan, Poland; dziental@ump.edu.pl (D.Z.); marcin.wysocki@student.ump.edu.pl (M.W.); michalakm500@gmail.com (M.M.); 4 Doctoral School, Poznan University of Medical Sciences, Bukowska 70, 60-812 Poznan, Poland; 5 Department of Engineering Fundamental Sciences, Sakarya University of Applied Sciences, 54050 Sakarya, Türkiye; eguzel@subu.edu.tr

**Keywords:** PDT, PACT, porphyrin, nanotechnology, bacterial infections, chlorin e6, bacteriochlorin, isobacteriochlorin

## Abstract

Nanotechnology is an emerging field that involves the development of nanoscale particles, their fabrication methods, and potential applications. From nanosized inorganic particles to biopolymers, the variety of nanoparticles is unstoppably growing, offering huge opportunities for drug delivery. Various nanoformulations, such as nanoparticles, nanocomposites, and nanoemulsions, have been developed to enhance drug stability, solubility, and tissue penetration. Moreover, nanocarriers can be specifically engineered to target diseased cells or release the drug in a controllable manner, minimizing damage to surrounding healthy tissues and reducing side effects. This review focuses on the combinations between porphyrin derivatives and nanocarriers applied in photodynamic therapy (PDT). PDT has emerged as a significant advance in medicine, offering a low-invasive method for managing infections, the treatment of tumors, and various dermatoses. The therapy relies on the activation of a photosensitizer by light, which results in the generation of reactive oxygen species. Despite their favorable properties, porphyrins reveal non-specific distribution within the body. Nanotechnology has the capability to enhance the PS delivery and its activation. This review explores the potential improvements that are provided by the use of nanotechnology in the PDT field.

## 1. Introduction

Photodynamic therapy is a low-invasive treatment method that uses a photosensitizer (PS), molecular oxygen, and appropriate light to kill cancer cells or microorganisms [1]. Despite its drawbacks, resulting from PDT principles (problems in PS excitation deep inside the diseased tissues), there are many approaches to overcome them. Some such protocols make PDT more invasive in comparison to their original form, i.e., the interstitial PDT, which requires the laparoscopic delivery of fiber optics with excitation light. In the context of anticancer therapy, the term PDT is used, while, in the context of antimicrobial therapy, there are two abbreviations, aPDT (antimicrobial photodynamic therapy) and PACT (photodynamic antimicrobial chemotherapy) [2,3]. The principle of PDT consists of the administration of a PS that is inactive in the dark and accumulates in the tumor, followed by local irradiation with the light of a wavelength corresponding to the PS absorption band needed for its activation. Simplifying the above, excited PS transfers its energy to molecular oxygen, thereby generating cytotoxic reactive oxygen species (ROS), such as singlet oxygen, which can oxidize key cellular components, leading to the destruction of cancer and bacteria cells [4]. Each ingredient is non-toxic and has no harmful effect on the organism, unlike chemotherapy drugs that can cause systemic toxicity [4]. Both modalities, PACT and PDT, despite their similar background, are distinguished by significantly different approaches and effects. The term “chemotherapy” in PACT is associated with low, although noticeable, toxicity without irradiation (dark toxicity) toward microbial cells, which is clinically insignificant. The activity of PS against microbes is potentiated by light activation and the turning on of the photodynamic process. Such a situation is a consequence of the PS structure for antimicrobial use—mainly cationic PSs, which enable the attachment to the bacterial membrane—at the microbial target site. Interestingly, in the case of mammalian cells treated with PS in the dark, the activity should not be noticed.

The key challenges for the photodynamic method development are associated with the proper targeting and delivery of the PS into the site of action. Another challenge is associated with limited tissue penetration by light, which is strongly dependent on the light wavelength, but also due to the absorption by the tissues and present chromophores, scattering, and refraction [1,5,6].

Unfortunately, PDT methods reveal some problems that need to be solved. Even with the use of NIR light, only tumors located no more than 1.5 cm deep under the skin can be destroyed because of poor light penetration [7]. Major limitations include neoplasia of unknown origin, the requirement for light, which precludes treatment of systemic disease, poor light penetration into extensive neoplasia, and damage to any major blood vessels in the vicinity of neoplasia. Due to local effects, PDT is ineffective in the treatment of metastatic lesions of unknown localization, which are the most common cause of death in oncological patients [8], and immunostimulation by PDT alone is not sufficient in these cases. The PS should possess most of the following features: hydrophilicity for easy systemic use, lack of dark toxicity, biocompatibility, the reliable generation of ROS after light irradiation, and targeted action [9]. In practice, there are often difficulties in achieving the above objectives.

**Figure 1:**
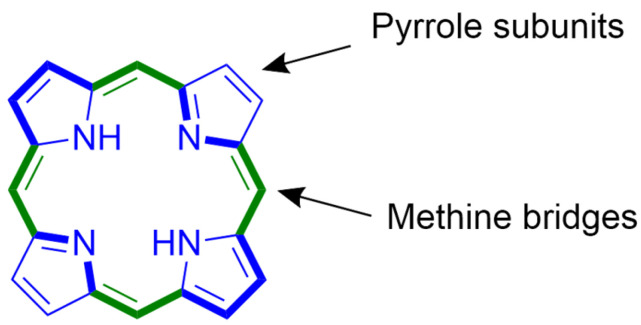
Basic structure of porphyrin with a bolded aromatic 18 π-electronic system.

Porphyrins (Figure 1) are a large group of compounds known for their important role in nature. After many years of research, porphyrins and their derivatives are currently one of the most frequently used PSs in photodynamic therapy (PDT). Their main advantages are the stable excited triplet state, the preferential accumulation in tumor tissues, the often high singlet oxygen-generation ability, and the broad absorption range, often from blue to red light. An additional factor is associated with the macrocyclic ring, which is prone to modifications by the insertion of metals that can enhance their properties [10,11,12,13].

Currently, PACT is mainly used in the treatment of superficial wounds, acne, and periodontitis [14]. It should be remembered that the bacteria killing at the level below 99.9% (<3 log reduction in bacterial growth) is clinically insignificant.

However, the path of application for porphyrins in photodynamic methods was not easy due to the low values of the absorption coefficients in the so-called “therapeutic window”, assigned to a range of 600–800 nm [15]. The results of any therapy depend not only on the chemical properties of the therapeutic agent but also on its affinity for tissues [16]. For this reason, attempts have been made to increase the solubility of porphyrins in aqueous solutions—where the first path is to modify the chemical structure—mainly by adding carboxyl, ester, ammonium, or sulfate groups [17]. Another method is the functionalization of porphyrins with bioactive molecules such as peptides, amino acids, antibodies, or vitamins. The third approach, which will be thoroughly analyzed in this work, is the combination of porphyrins with carriers. Since the carriers are prone to modification, they seem to be the most versatile solution for enhanced targeting due to appropriate moieties present on their surface. Proper localization and the preferable accumulation in the tumor allow for the minimization of the side effects of the therapy, as well as providing more effective delivery into the site of action. Pharmacokinetic adjustment to make it more convenient is also a crucial factor that is often taken into account. Additionally, the carriers can be combined with other specific moieties that ensure the controlled release under the desired conditions, such as the specific pH. The possibility of equipping the nanocarriers with other drugs can also enhance the effectiveness of the used therapy due to the multifunctional approach. Concerning the properties of porphyrins, such an approach can effectively overcome their limitations [1,6,17].

## 2. Combinations of Porphyrins with Liposomes

**Figure 2:**
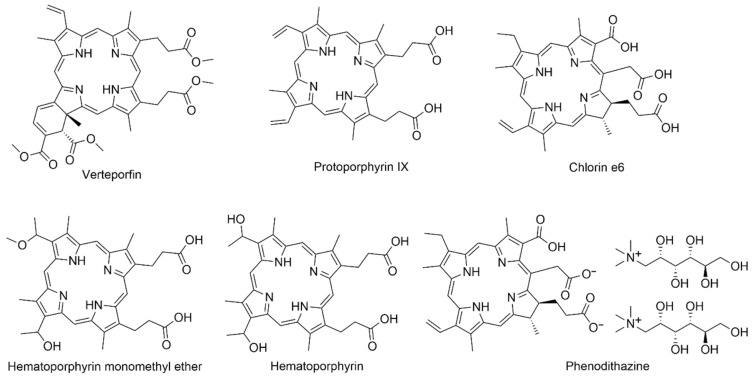
Common derivatives of natural porphyrins used as drugs.

The use of liposomes to transport hydrophobic molecules improves their solubility in an aqueous environment and thus reduces their tendency to aggregate. Additionally, the surface of liposomes can be relatively easily modified to increase drug accumulation in cancer cells [18,19]. The first drug containing porphyrins in combination with liposomes approved by the FDA was benzoporphyrin, with the trade name Verteporfin^®^ (Figure 2), also known under the trade name Visudyne^®^.

**Figure 3:**
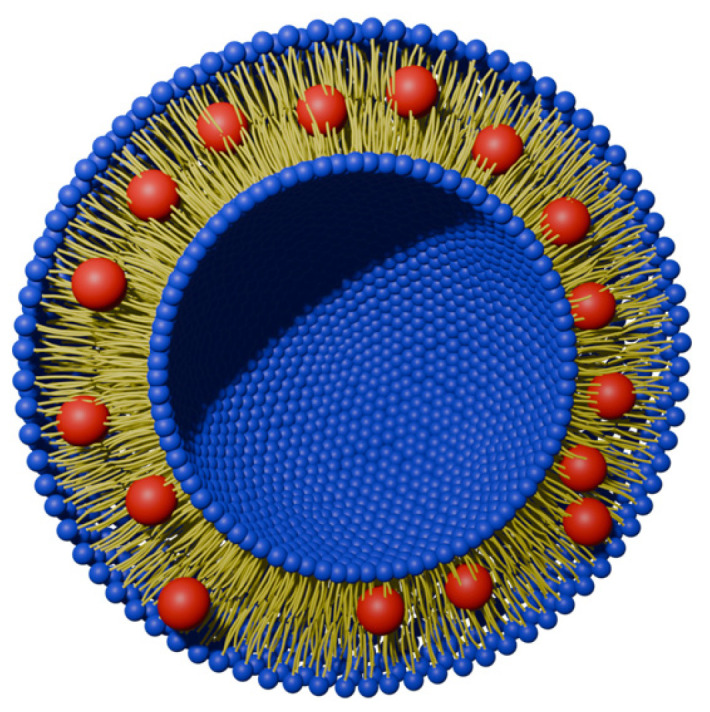
Internal structure of the liposome loaded with hydrophobic PS (red).

It is currently used to treat age-related macular degeneration (AMD) and subfoveal choroidal neovascularization occurring in high myopia [20]. The product consists of a benzoporphyrin derivative and a liposome (Figure 3) composed of unsaturated phosphatidylglycerol and dimyristoylphosphatidylcholine in a molar ratio of 3:5 [21]. Since non-ionic porphyrin derivatives are poorly soluble in water (except for PEG-ylated ones), and thus susceptible to self-aggregation in such an environment, this results in a decrease in their efficiency due to the self-quenching phenomenon [22]. The use of the formulation enables the better uptake of the PS into the target tissues. Moreover, the ease of their modification allows for the implementation of drug release in the presence of a specific trigger, such as a low pH, the presence of certain enzymes, an oxidant (generated singlet oxygen), or heat [23,24,25]. Despite all of the benefits, the liposomal formulations have also drawbacks, such as low in vivo stability, the possibility of lipid peroxidation, rapid drug release, and delivery to the particular subcellular organelles. Additionally, their limited stability creates problems with long-term storage and is associated not only with their degradation, but with the modulation of the physicochemical properties of the formulations by degradation products as well [26]. The drawbacks of liposomal formulations can be overcome by utilizing strategies that rely on incorporating stability enhancers, such as functional polymers, that can also provide a more controlled release. Moreover, the inclusion of targeting moieties should improve the delivery precisely into cancer cells. The polymeric moieties themselves, despite beneficial properties, are burdened with limited drug capacity [27]. Thus, by creating hybrid polymer–lipid particles (PLHNPs), it is possible to obtain favorable features of both components, such as high biocompatibility, improved incorporation of the drug into the carrier, controlled drug release, and increased drug accumulation in the target tissue [27]. Additionally, the conjugation of lipid nanoparticles, e.g., with folic acid or proteins (such as antibodies), is also used to increase the effectiveness through an active delivery system directed at the folate receptor or epithelial growth factor receptors (EGFRs), which is a targeted therapy [27,28,29].

Similarly to anticancer applications, liposomal formulations have been successfully utilized against microbes. The key difference between those two aspects is that the most active PSs against microbes possess cationic groups, although there are many exceptions regarding the high concentration of anionic and non-ionic PSs. Furthermore, the differences in the bacterial outer membrane also determine the PS efficacy. Gram-negative bacteria are often less vulnerable to PDT due to the thick membrane, contrary to Gram-positive bacteria [30]. As in the case of the anticancer PDT, the majority of porphyrin PSs were neutral, and thus hydrophobic, so they were incorporated into the lipid bilayer. On contrary, antibacterial cationic porphyrins are more hydrophilic and can accumulate in the aqueous core [30]. PSs delivered in liposomes most often demonstrate higher photodynamic effectiveness against bacteria than free PSs [31]. Ferro et al. [32] developed a new, positively charged *meso*-substituted porphyrin—5-[4-(1-dodecanoylpyridinium)]-10,15,20-triphenylporphyrin (Table 1, no. 11), characterized by very efficient ROS formation. Nevertheless, it showed relatively low bactericidal activity (below 3 log) when dissolved in a homogeneous aqueous solution or incorporated into neutral lipid vesicles. The effect was enhanced when polycationic agents were used as carriers, such as liposomes containing N-[1-(2,3-dioleoyloxy)propyl]-N,-N,-N-trimethylammonium chloride. It acted primarily as an agent that disorganized the structure of the bacterial cell wall, and, as a consequence, the drug could penetrate deep into the cell membrane and quickly weaken the selected enzymatic activities, leading to cell death. A reduction in bacterial growth of about 4.5–6.0 log was achieved [32]. The study was conducted on a strain of MRSA [33]. The severity of these infections varies from minor skin infections to fatal necrotizing pneumonias [33]. The combination of cationic delivery systems and positively charged drugs is an innovative method of improving the effectiveness of classic PSs, enabling the use of PACT on a larger scale [32]. Tsai et al. [34], in their study on *S. aureus*, *Staphylococcus epidermidis*, and *Streptococcus pyogenes*, investigated the combination of hematoporphyrin IX (Figure 2) with liposomes. They demonstrated that the use of cargo could induce a high rate of bacterial eradication (4–5 log) at a much lower dose than that in the case of application of the porphyrin alone.


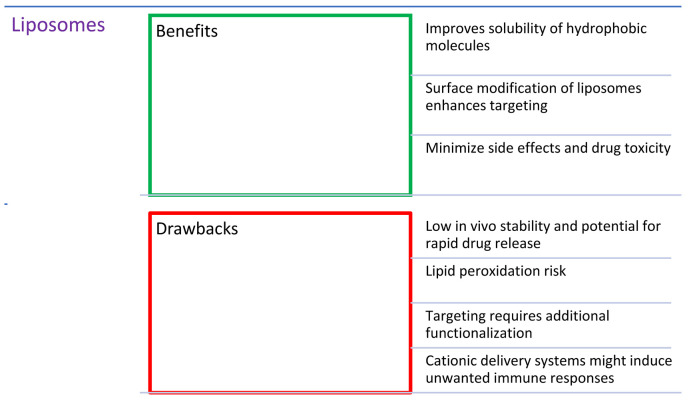


## 3. Combination of Porphyrins with Polymers

**Figure 4:**
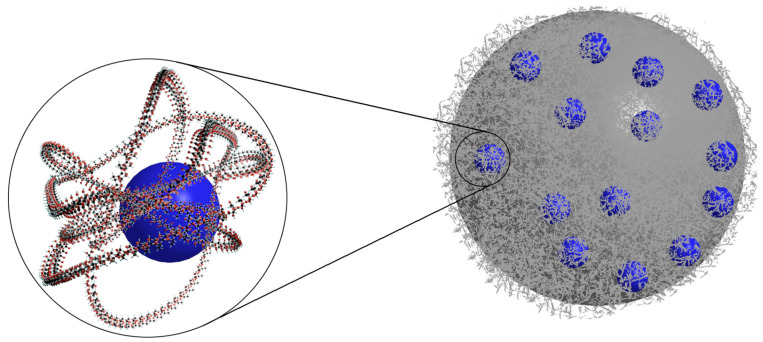
Visual representation of the PS (blue) incorporated into the PLGA NP.

Many scientists have become interested in the potential of PLGA (a copolymer of lactic and glycolic acid; see Figure 4) nanoparticles due to the specific and controlled delivery of various micro- and macromolecules, including drugs [16]. In the formulation of various polymer nanostructures, PLGA has gained attention because of its properties such as complete biodegradability and biocompatibility, well-understood formulation techniques, and easy modification [16]. Its huge advantage is the easy functionalization of polymers, enabling the creation of structures tailored to porphyrins of different molecular weights, hydrophobicity, and charge. In this way, the biodistribution and kinetics of the drug can be optimized [48]. Since PLGA is fully hydrophobic, contrary to earlier mentioned liposomes, where only the lipid bilayer shares the hydrophobic nature, the encapsulation of non-functionalized porphyrins in the NPs can be more effective than in case of liposomes of a similar size [49]. To further improve the efficacy of drug delivery and the therapy itself, the surface of the PLGA nanoparticles (NPs) can be modified with specific proteins such as lectins. This kind of modification may greatly enhance the targeting due to high specificity toward sugar moieties expressed on the membrane and organelle proteins, similarly to liposomes, as mentioned earlier [50]. Interestingly, there is a possibility to coat the PLGA NPs with lipids, creating a huge liposome-like structure with the loaded PLGA NPs [51]. Additionally, thanks to nanoencapsulation, the effectiveness of the complex is improved, allowing for a lower dose and, consequently, a reduction in the side effects caused by porphyrins without losing the effectiveness of the treatment [48]. On the other hand, formulations based on PLGA possess some drawbacks—their biodegradability is related to rapid hydrolysis, which can limit its application in aqueous media. The strategies used to achieve better durability in the organism involve changing the lactic acid/glycolic acid ratio or the incorporation of other polymers or ceramic scaffolds. Since both monomers have different properties—poly(glycolic acid) is crystalline and less hydrophobic, while poly(lactic acid) is amorphous and more hydrophobic—this allows for the effective tailoring of the hydrolysis rate, as there a higher content of poly(lactic acid), although it should be remembered that the composition in 1:1 ratio is most susceptible to hydrolysis, and therefore it degrades at the highest rate [49].

**Figure 5:**
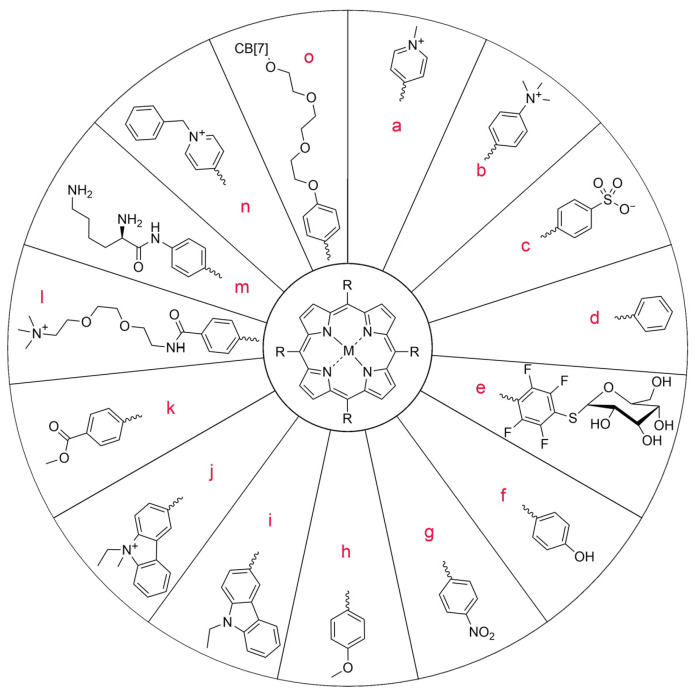
Modifications of symmetrically substituted (A_4_-type) porphyrin described in this paper.

In a study by Gonzales-Delgado et al. [52], a highly water-soluble PS, 5,10,15,20-tetrakis (1-methylpyridinium-4-yl)-porphyrin tetraiodide (TMPyP; Figure 5a), was enclosed in PLGA. Free nanoparticles were introduced into the Carbopol hydrogel to achieve a controlled release of porphyrin. This formulation was tested on a model analogous to the permeability profile of skin, a pig ear skin model. No penetration into the deeper skin layers and satisfactory effectiveness at the site of administration were demonstrated. This property makes the combination of encapsulated PSs with carriers on gel bases enabling modified release very promising [52]. Ito et al. [53] focused on a similar aspect in their work using manganese–TMPyP (Figure 5a). They confirmed the relationship between the molecular weight and the effect of the drug on the tissue and the potential improvement of the treatment effectiveness in PDT. The smallest particles showed the greatest loading effectiveness but also the fastest penetration into tissues, which is the desired effect.

**Figure 6:**
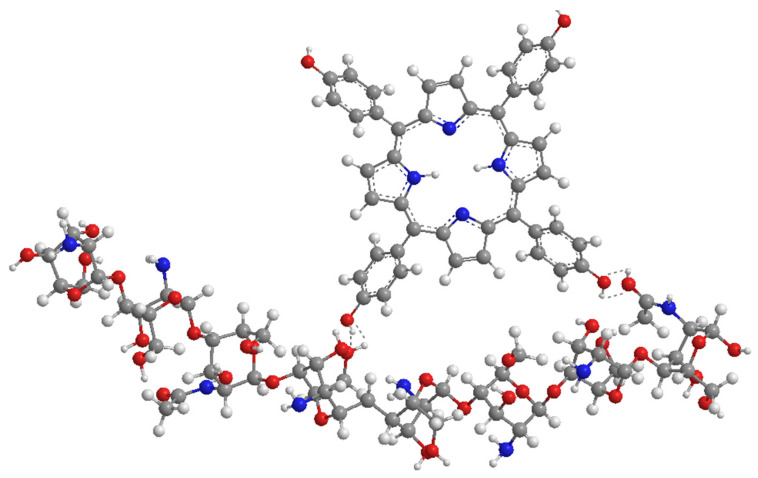
Structural fragment of chitosan with porphyrin bonded via hydrogen bonds.

An interesting option, lately gathering more and more attention, are carriers based on chitosan. Chitosan (Figure 6) is a natural carbohydrate polymer obtained from mushrooms and crustacean shells by the partial N-deacetylation of chitin [54]. It has unique mucoadhesive properties, which means it adheres very well to wet surfaces inside and outside of the human body. Thanks to this, it is used in MDDSs (mucoadhesive drug delivery systems). Its wide use in medicine results from its high biocompatibility and lack of documented allergic response [55]. Chitosan contains heteroatoms in its structure that can form hydrogen bonds with porphyrins, and its lipophilic properties enable the transfer of the complex to the organic environment [55]. Moreover, due to the presence of amine groups, it can effectively bind the PS molecules via imine (Schiff base) or amide bonds, as well as additional alkyl chains. Amine groups are also useful for cross-linking, allowing for the easy conversion of chitosan into hydrogels, which often present excellent self-healing properties and long-term stability [56,57,58,59]. Overcoming both hydrophilic and hydrophobic drug obstacles makes chitosan suitable for use in combination with many molecules. It has also been proven that it can enhance the therapeutic effect due to the high adsorption rate on phospholipid monolayers—analogous to the cell membrane [60]. On the contrary to liposomes and PLGA, chitosan can be soluble in water, although it is highly dependent on its molecular weight, which poses a problem. This can be improved by lowering the pH due to the protonation of amino moieties. Another disadvantage of chitosan is its low mechanical strength, which is possible to overcome with the incorporation of other materials, such as polymers [57,59].

Chitosan (CS) is also used as a carrier in PACT for the following three main reasons: its antibacterial properties, the positive charge of the molecules, enabling them to penetrate or bind to the bacterial cell membrane, and thanks to its ability to increase hydrophilicity [61,62]. Gsponer et al. [63] used a porphyrin–chitosan hydrogel to inactivate *E. coli* with different CS concentrations. In the dark conditions, a decrease in the cell number was observed, especially at higher concentrations, indicating the cytotoxicity of chitosan itself. It has antimicrobial effects because its positive charge can disrupt the functions of the negatively charged membrane, changing its permeability [63]. Thus, a low CS concentration resulted in decreased phototoxic activity induced by 5,10,15,20-tetrakis (4-N,N,N-trimethylammoniophenyl)porphyrin (TMAP^4+^; Figure 5b) from ca. 7 to 5 log, probably due to the repulsion of positive charges. Nevertheless, this reduction is still more than enough. On the other hand, CS enhanced the photoinactivation of *E. coli*, with the 5,10,15,20-tetra(4-sulfonatophenyl)porphyrin (TPPS^4−^; Figure 5c) and the reduction in bacterial growth increasing from ca. 1.5 to 4 log [63].

**Figure 7:**
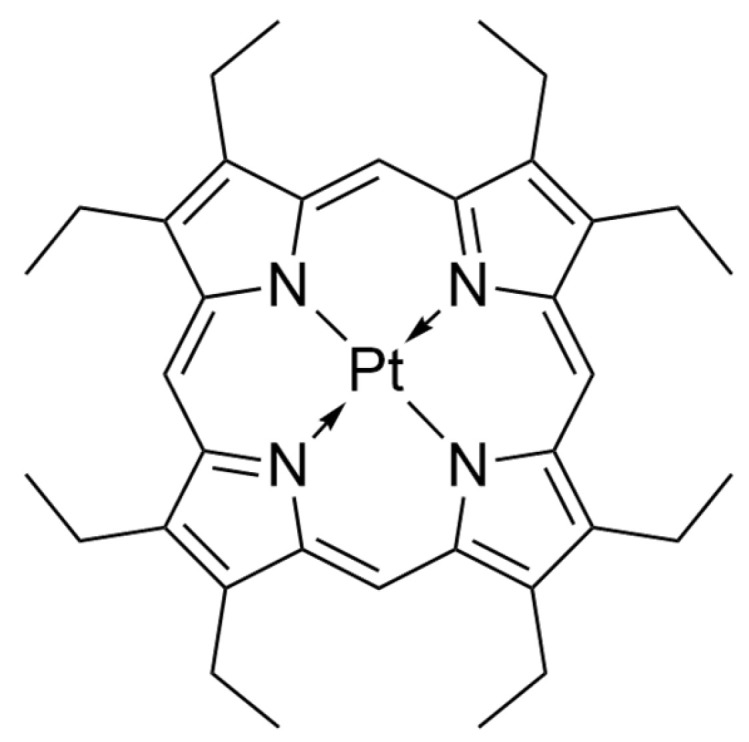
The structure of non-*meso*-substituted platinum(II) octaethylporphyrin.

Polymer nanoparticles have gained popularity in the context of use in PACT mainly due to minimizing the precipitation or aggregation of hydrophobic PS molecules in a polar environment and enhancing the interaction with the target cell [31]. Their incorporation may result in formation of polymeric vesicles, polymersomes, that exhibit interesting behavior. Their membranes are able to allow singlet oxygen through without their disruption, which is not always available with liposomes [64]. Martinez et al. [65] tested a combination of polymer and platinum octaethylporphyrin (Figure 7) on antibiotic-resistant bacteria from the ESKAPE pathogen group. The following bacteria strains belong to the group: *Enterococcus faecium*, *S. aureus*, *K. pneumoniae*, *A. baumannii*, *P. aeruginosa*, and *Enterobacter* spp. The rates of bacterial growth achieved values from 3 to 6 log. They are able to avoid the action of antibiotics and have developed new resistance mechanisms [66,67]. Therefore, the research aiming to find an effective way to eradicate these pathogens is particularly important. It was demonstrated that, among them, Gram-negative bacteria required a higher dose of light (by approximately 10–15 J/cm^2^) to exert the desired effect, but they were equally successfully removed from the biofilm [65].

Khelifa et al. [68] compared the activity of *meso*-phenylporphyrin (Figure 5d) in relation to its polymer derivative. It turned out that *meso*-phenylporphyrin has moderate activity against *E. faecalis*, with an inhibition zone, as measured by the disk-diffusion method, of 11 mm and low activity against *S. aureus*, with an inhibition zone of 4 mm. However, inhibition was not observed for *P. aeruginosa* and *E. coli*. The polymer conjugate showed much higher antibacterial activity against all of the tested strains—the inhibition zones were 11 mm for *P. aeruginosa*, 12 mm for *E. coli*, 14 mm for *E. faecalis*, and 15 mm for *S. aureus*, respectively. These values are comparable or slightly higher than those obtained for tetracycline, which causes a zone of inhibition of *P. aeruginosa* and *E. coli* of approximately 12 mm in diameter.

Modern drug formulations, such as hydrogels, are increasingly used in biomedical applications. Their unique physical properties provide a different approach to PS delivery in PACT, especially for wound infections [69]. The hydrogel combined with porphyrin is able to adapt to the shape of the wound and effectively release PS, which makes PACT take place in a controlled manner under exposure to light [69]. After the treatment, the expanded structure of the hydrogel allows for easy removal. These features may facilitate the clinical application of PACT. Donnelly et al. [69] prepared a poly(vinyl alcohol) (PVA) hydrogel containing methylene blue and porphyrin *meso*-tetra-(N-methyl-4-pyridyl)-tetra-tosylate (Figure 5a). The conjugate showed a high effectiveness in PACT and the potential for use as a novel approach to the treatment of wound infections [69].


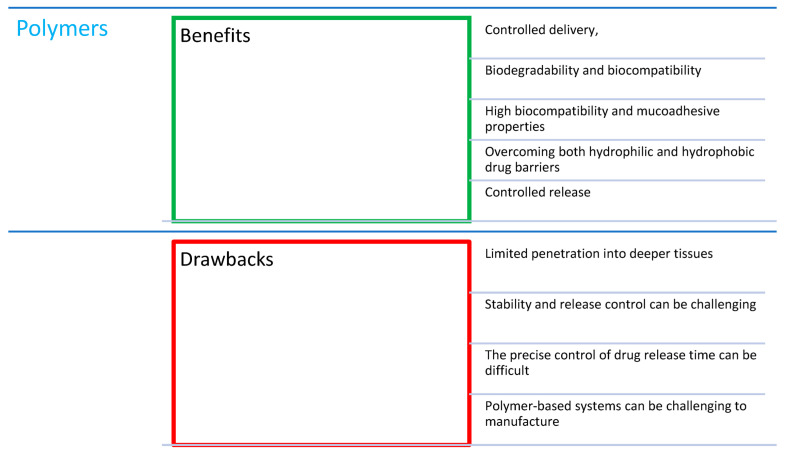


## 4. Combination of Porphyrins with Virus-like Particles

**Figure 8:**
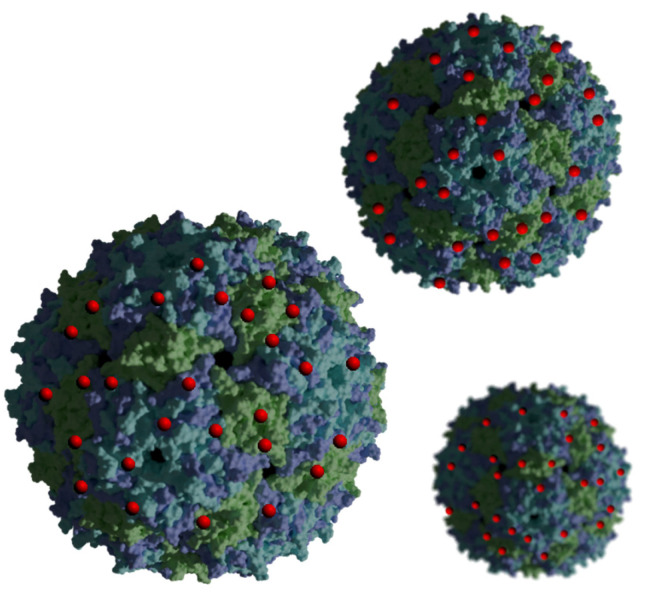
Appearance of virus-like particles carrying PSs (red). Generated in Blender 4.1 using PDB data [73,74] and modified.

Virus-like nanoparticles (VLPs; Figure 8) are another path in the discovery of new nanocarriers in PDT. These particles possess many advantages, including a low price, natural origin, and the ability to carry a large amount of drug compared to other nanoparticles [18,70]. Moreover, they are non-infectious particles based on a protein structure, so they can naturally react with numerous types of receptors present on the cell membrane, and thus they can cause the intended effect in the cell [18]. It is worth mentioning that VLPs non-equipped with targeting moieties can provide no effect; thus, introducing proper targeting ligands may be crucial to achieving the desired activity. VLPs are also relatively easy to link to porphyrins and attach various molecules to the surface due to vast amount of various functional groups that can be utilized either to create linkers or serve as one. Porphyrins are enclosed in VLPs through covalent and non-covalent bonds [70,71]. Non-covalent bonds are easier to obtain, while covalent bonds have the advantages of providing effective encapsulation and improving stability. Interestingly, the possibility of expression of specific peptide sequences or proteins also exists and is associated with genetic methods of capsid modification. The modification of surface groups by covalent bonds is likely to follow standard reactions used in organic chemistry, and the introduced moieties may include the following popular functional groups: phenol, thiol, amine, azide, carboxyl, and others. Such a conjugation provides high stability of the cargo [70]. However, VLPs have a lower toxicity than inorganic particles, a higher stability compared to liposomes, and are more homogeneous compared to polymer particles [72].


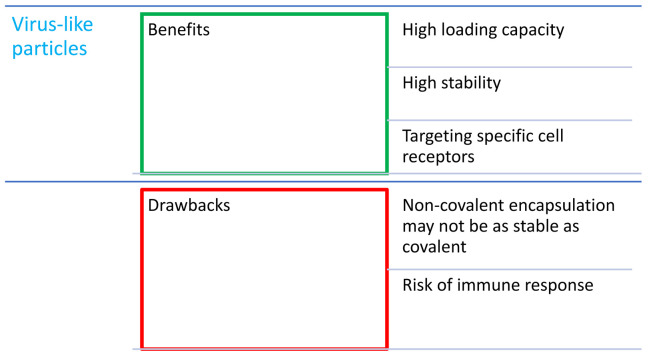


## 5. Combinations of Porphyrins with Hydrocarbonates

**Figure 9:**
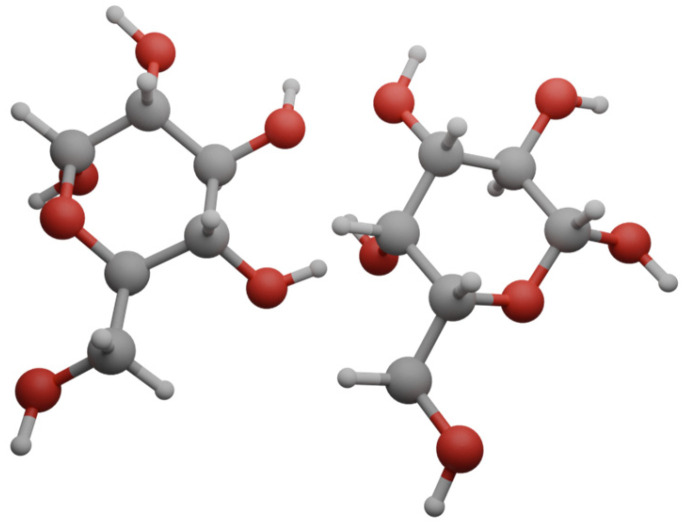
Glucose and galactose molecules.

Cancer cells have a high energy demand and their proliferation is often dependent on glucose uptake. Therefore, it was decided to investigate the effect of combining porphyrins with sugars (Figure 9) as selective delivery enhancers in PDT. Cancer cells overexpress glucose transport receptors, so porphyrin–saccharide bioconjugates should reveal a higher binding affinity than porphyrins alone [75]. Interestingly, conjugation with sugar moieties can improve the uptake of poorly water-soluble PSs. An important targeting factor is also the overexpression of lectin-type receptors, which are specific for glycosylated structures. The greatest problem in combining porphyrins with saccharides is the hydrolysis of the sugar component attached via O-glycosidic bonds under physiological conditions, which leads to the short half-life time of these molecules in the organism [75,76]. However, porphyrins with sugars attached by S- or C-glycosidic bonds showed low synthesis yields, so many studies have focused on improving the synthesis methods [75,76]. An additional difficulty is also the aggregation in aqueous solutions, where PS aggregates are formed, for which a decrease in singlet oxygen generation is observed [76].

In a study published by Singh [77] et al., the aggregation of tetra-thioglycosylated porphyrins (Figure 5e), chlorins, and bacteriochlorins was compared. Despite the formation of particles of different sizes, all of them were captured by cancer cells, which was proven with fluorescence microscopy. The porphyrin-incorporated polymer, which was enriched with galactose molecules, revealed lower IC_50_ values for HepG2 and Hep2 (0.2 mg/mL and 0.4 mg/mL, respectively) compared to the polymer without galactose, for which the IC_50_ values were higher (0.5 mg/mL and 0.5 mg/mL, respectively, for each carcinoma cell line) [78].

The structural organization of sugar nanoparticles with porphyrinoids depends on many factors, including the number of attached sugar groups and the type of component molecules, the bond type, the concentration, the solvent, and the mixing technique [79]. From all of the above-mentioned studies, it can be concluded that the potential effectiveness of saccharide–PS conjugates in PDT depends mainly on the type of sugar moiety and the photodynamic profile of the porphyrin, as well as the place and nature of the sugar attachment.

**Figure 10:**
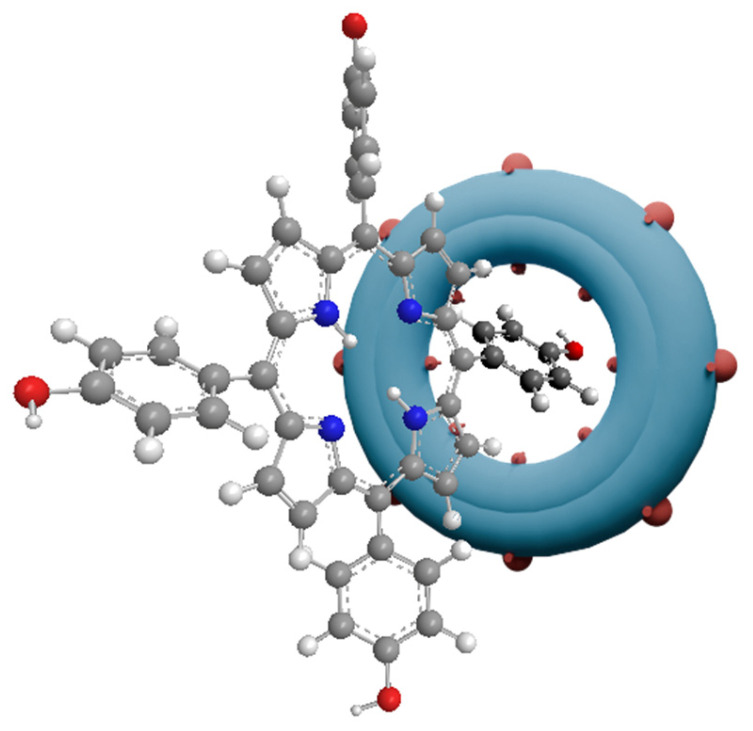
Visual representation of β-cyclodextrin (blue ring) associated with the peripheral group of porphyrins.

Cyclodextrins (CDs) are macrocyclic carbohydrate compounds containing six, seven, or eight glucopyranose rings connected by α-1,4-acetal bonds, which have the ability to incorporate hydrophobic compounds into their cavity by forming inclusion complexes (Figure 10) [80]. This property makes cyclodextrins an ideal candidate for combinations with porphyrins. CDs increase the hydrophilicity of the conjugate and prevent particle aggregation. The association of porphyrins with cyclodextrins also brings many benefits. CDs can attach moieties that target specific organelles and be functionalized with compounds for imaging purposes. The possibility of immobilizing drug molecules makes the cyclodextrin carrier suitable for treatment with photochemical internalization—an innovative drug delivery mechanism involving the simultaneous delivery of a chemotherapeutic agent, a PS, and light [80]. An obstacle to the use of cyclodextrins is the high concentration required (>10%) to ensure the appropriate formulation of the drug [81]. Therefore, another component was planned to be added to the conjugates—polymers. In a study by Bergh et al. [81], 5,10,15,20-tetrakis (4-hydroxyphenyl)porphyrin (THPP; Figure 5f), *β*-cyclodextrin derivatives, and a copolymer—poloxamer 407—were used. It turned out that the solubility of THPP increased 850 times in binary systems containing *β*-CD and 10,000 times in three-component systems with a copolymer [81]. In the next study, these conjugates were tested in the treatment of infected wounds [82]. Open wounds that become infected by bacteria often last a very long time and are difficult to treat, so a drug formulation containing porphyrins administered directly to the wounds would be a very good therapeutic option, especially since bacteria do not tend to acquire resistance to them [82]. The base substance in the foam (the drug form was chosen due to more precise dosing than in the case of gels or creams) [82] was alginic acid, which is a natural copolymer of mannuronic acid and glucuronic acid. Alginate-based dressings are widely used in wound treatment, as they have the ability to effectively absorb exudate, thus providing an optimal environment that promotes healing. The drug release from the tested foams was tested and adjusted by the amount of CD in the formulation and the degree of its cross-linking. This allows for the design of a rapid or sustained-release formulation [82].

As far as cyclodextrin is concerned, its presence, as reported Hanakova et al. [83], cannot be beneficial for the activity. They tested cyclodextrin combined with zinc(II) tetrakis(4-sulfonylphenyl)porphyrin (Figure 5c) against the Gram-positive bacteria *S. aureus*. After irradiating bacterial cultures with a relatively high light dose of 150 J/cm^2^ and at a relatively high PS concentration of 100 µM, cell viability was reduced by only 92% (ca. 1 log reduction) [84].

**Figure 11:**
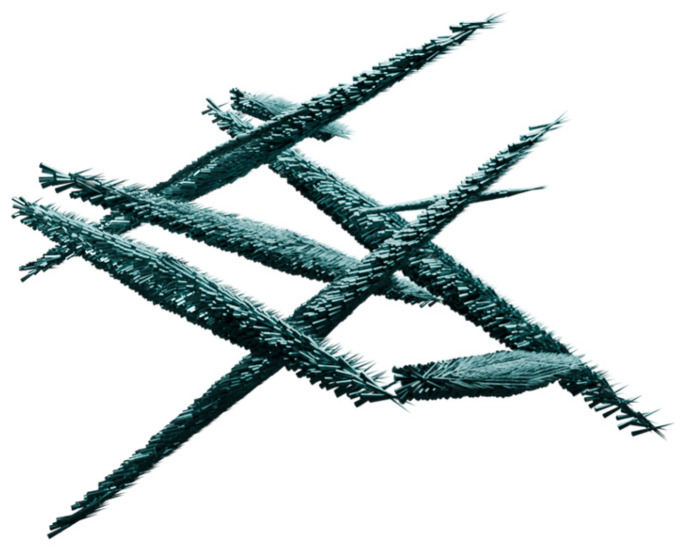
Visual representation of cellulose NPs.

Cellulose, a hydrocarbonate polymer, has extremely useful physicochemical properties, such as hydrophilicity, biocompatibility, biodegradability, and a large specific surface area of particles (Figure 11), which have been used in PDT by combining cellulose with porphyrins, as shown by Rahimi et al. with an A_4_-type porphyrin (Figure 5b) [85]. The structure of cellulose NPs consists of many chains fitted together more or less orderly, resulting in the presence of crystalline and amorphous domains, respectively. Those domains possess different properties, so they can be separated. The amorphous units break down, while the crystalline ones remain stable, forming nanocrystals used further for various applications [86]. Similarly to earlier-mentioned chitosan and VLPs, the cellulose chains can be modified by subjecting the hydroxyl groups to chemical reactions, resulting in their esterification, oxidation, or substitution

In a study conducted by Fayyaz et al. [87], nanocellulose (NCN) crystals were formed and meso-tetrakis(4-nitrophenyl)porphyrin (TNPP; Figure 5g) was incorporated. Then, the photoinactivation of *Bacillus subtilis* and *Pseudomonas aeruginosa* bacteria on agar was examined. The resulting conjugate revealed negligible activity against *P. aeruginosa* in comparison to *B. subtilis*. The inhibition of bacterial growth was complete within the inhibition zone for *P. aeruginosa*, while occasional growth was observed in *B. subtilis* colonies within these zones, clearly indicating that *B. subtilis* is naturally more resistant to PACT. The binding percentages of porphyrins to *B. subtilis* and *P. aeruginosa* in this study were 1.89% and 16.78%, respectively. The percentage of binding of *P. aeruginosa* to TNPP is higher than for the other strains, which may justify its greater effectiveness. Previously, Fayyaz et al. tested the combination of NCN with another porphyrin, tetrakis(4-N,N,N-trimethylanilino)-porphyrin (Figure 5b), demonstrating more effective photoinactivation. This can be attributed to the better solubility and more effective binding of porphyrin to bacteria [85]. Carpenter et al. [41] demonstrated the promising antimicrobial activity of nanocellulose crystals combined with an A_3_B-type porphyrin (Table 1, no. 7) against cultures of *Acinetobacter baumannii* (5–6 log reduction) and MRSA (methicillin-resistant *Staphylococcus aureus*—6 log reduction) after irradiation with visible light. Imaging showed PS fluorescence mainly outside of the *A. baumannii* and *S. aureus* cells, suggesting that the cellulose nanocrystal bound porphyrin remained covalently bound to the cellulose during the cell irradiation test and was not degraded. As a result, reactive oxygen species generated in the photodynamic process are likely to spread over short distances, ultimately damaging the bacteria and leading to inactivation or cell death [41].


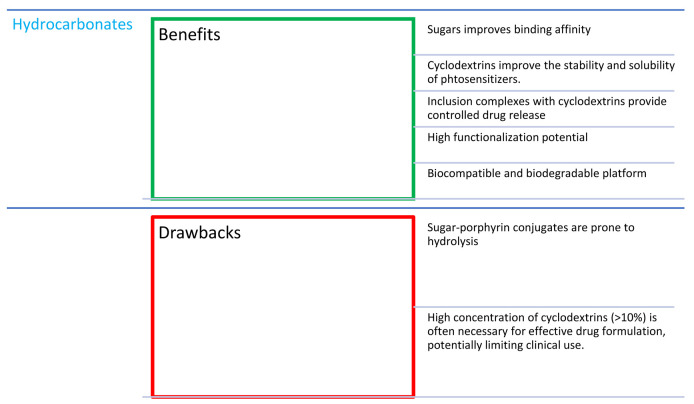


## 6. Combinations of Porphyrins with Dendrimers

**Figure 12:**
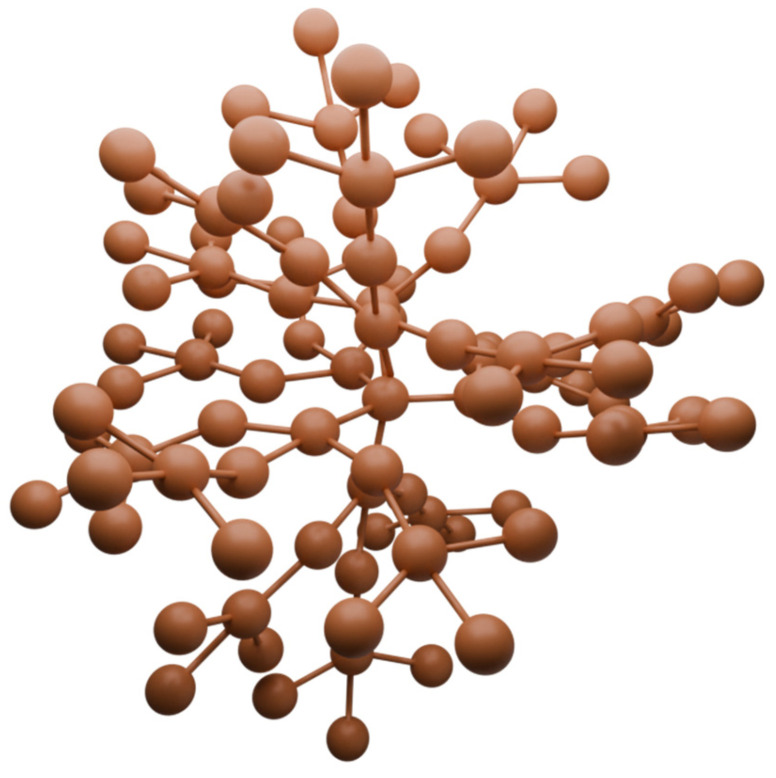
Visualization of the dendrimeric structure.

Dendrimers are molecules that contain symmetrical branched units built around a small molecule or linear polymer core (Figure 12). This term means only a structural arrangement, not the name of a specific compound [88]. Dendrimeric systems are very widespread in nature. They can be encountered from the meter scale in tree branches, through the millimeter scale in human anatomy, to the molecular level, where this system is represented, among others, by glycogen and proteoglycans [89]. These hyperbranched macromolecules can be functionalized relatively easily, thereby modifying their physicochemical or biological properties. This and many other features have led to their use as “nanoscale delivery devices” [88]. Biologically active molecules can be placed inside the dendrimers or adsorbed on their surface, forming two types of porphyrin–dendrimer connections [89]. The advantages of dendrimers are the following: a high loading capacity and the ability to modify the lipophilicity and size of the conjugate to optimize cellular uptake [90,91]. Among dendrimers, polyamidoamines (PAMAMs) are especially popular due to such features as low immunogenicity, good solubility, biodegradability, and precise structure, and, when combined with a glycosylated core, the selectivity of the conjugate is also improved [92].

In their study, Chang et al. [92] used glycosylated PAMAM dendrimers that specifically recognized lectin receptors. Cancer cells usually overexpress protein or glycoprotein receptors, including lectin ones. Thanks to the ability of the drug to recognize these receptors, high tissue selectivity and good singlet oxygen production efficiency are achieved. Militello et al. [93] used a combination of tetra-*p*-methoxyphenylporphyrin (Figure 5h) with PAMAM-class dendrimers and proved that the spectroscopic properties are determined by the porphyrin core of the molecule. Additionally, a low level of self-quenching was recorded, which is a desirable property in PDT. This results partly from the low molecular aggregation due to the steric hindrance of dendritic branches [93].


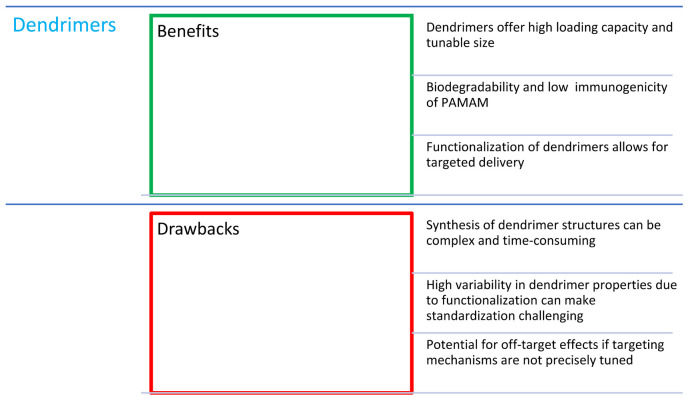


## 7. Combinations of Porphyrins with Carbon Nanoparticles

**Figure 13:**
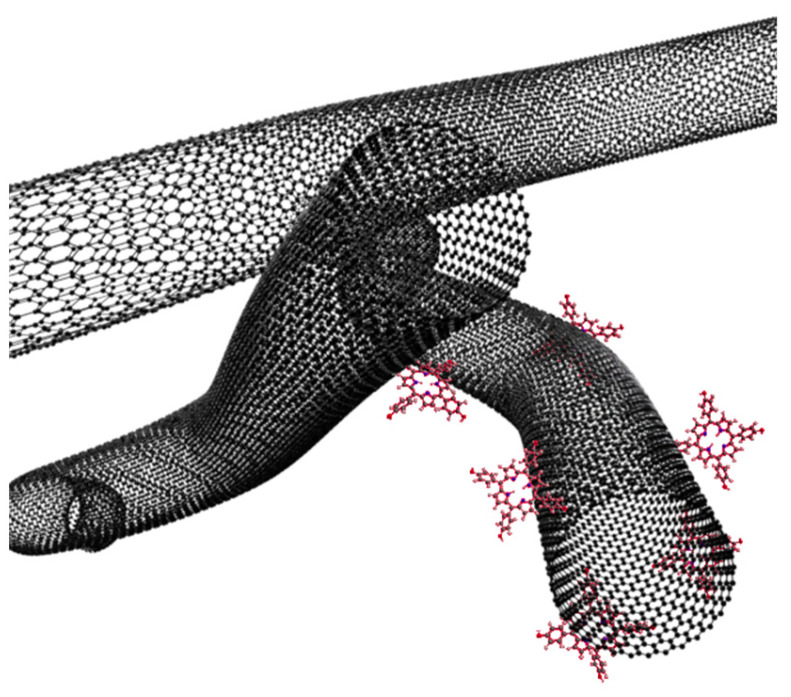
Visualization of carbon nanotubes with covalently attached PS.

Carbon nanotubes (CNTs; Figure 13) are cylindrical particles made of one or more layers of graphene with open or closed ends. They are divided into single-walled ones, SWCNTs (single-walled carbon nanotubes), and multi-walled ones, MWCNTs (multi-walled carbon nanotubes) [94]. Carbon nanotubes have many useful features, including a small size (with a length of a few mm and a diameter of approx. 1–50 nm), an elongated shape, the potential to carry large amounts of drug, a high tensile strength, high electrical and thermal conductivity, and susceptibility to modifications [95].

Porphyrins can be immobilized onto nanotubes using covalent or non-covalent bonds. In covalent bonds, surface of CNTs is firstly functionalized and then porphyrin-modified, resulting in stable bond formation. The structure of CNTs allows for radical-based reactions, and thus the attachment of polymeric chains containing a high content of porphyrin via the radical polymerization of its monomeric side groups (such as acrylate) is also possible [96,97]. Interestingly, SWCNTs themselves can generate singlet oxygen due to the transfer energy on oxygen atoms, promoting triplet–singlet transition. Such a phenomenon appears to have a synergistic effect when combined with porphyrins [98]. In a study by Wang et al. [38], the NLO (non-linear optic) properties of the SWCNT-TPP (carbon nanotube combined with A_3_B-type porphyrin; Table 1, no. 4) and SWNCNT-TPPZn (with zinc salt of the compound) nanohybrids were checked. Both nanohybrids showed better NLO performance compared to individual TPP, TPPZn, and SWCNT, which confirms the synergistic effect of the ingredients. Moreover, nanotubes are often combined with PLGA or hyaluronic acid, as well as modified with surfactants or proteins, to increase their solubility in an aqueous environment [95,99].

However, the promising properties of CNTs are associated with safety concerns. CNTs are likely to contain residues of catalysts and contaminants after the manufacturing process, which may significantly increase the toxicity via direct damage to the DNA in the affected cells. The toxicity of CNTs is highly associated with their physicochemical properties [100].

**Figure 14:**
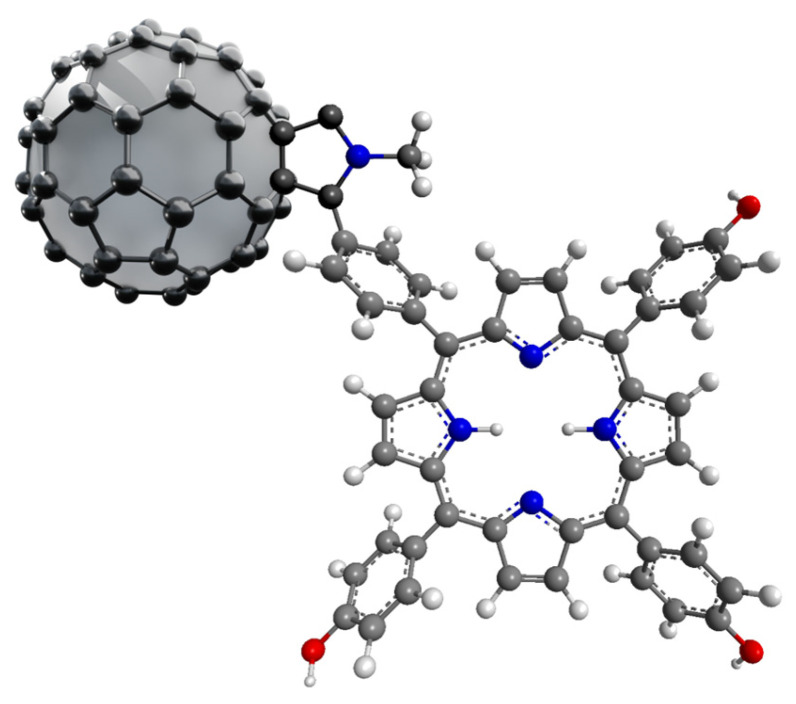
The visualization of C60 fullerene with covalently attached PS.

Fullerenes were discovered in 1985, and since then, their potential has been continuously explored in various fields of science, including medical applications. The unique structure of fullerene consists of biologically inert molecules (containing 60–100 carbon atoms) arranged in the structure of a football (Figure 14) [101]. Combinations of fullerene C_60_ with porphyrins are insoluble in water and show a high degree of aggregation in this medium, which is indicated as a main disadvantage of this carrier system. The mentioned problem can be solved by the use of chemical modifications of the fullerene surface [102]. On the other hand, the hydrophobicity of fullerene favors its greater affinity for cell membranes. The combination of porphyrin compounds with amino acids and fullerene peptide derivatives is designed to increase its solubility and the absorbable dose of the PS [101]. Due to their unique structure, fullerenes are able to accept up to six electrons [103], and, when exposed to light (UVA), these electrons move to a higher energy level, creating an excited singlet state *C_60_, which reacts with molecular oxygen to form singlet oxygen. Successively, fullerene changes from the singlet excited state to the triplet excited state, which is then able to generate superoxide anion radicals after combining with oxygen [104]. Studies have shown that there are significant differences between the long-known, hydrophobic, insoluble C_60_ fullerenes and the “aqueous” nC_60_ suspension prepared under appropriate conditions and using proper techniques [101]. Fullerenes, which dissolve more easily in water, are believed to contain residual solvent portions within the aggregates, which may modify their biological behavior. These solvents are, in some cases, chemically bonded to the molecule rather than merely adsorbed onto its surface [101].

The combination of porphyrins with fullerene makes the formulation more efficient in terms of the production of ROS [103]. The main direction of the development are their combinations with other molecules.

Fullerenes are molecules with strong antibacterial properties [103]. Many papers describe their effect on the inactivation of various bacterial strains [105,106,107]. Ballatore et al. synthesized a porphyrin–fullerene conjugate with three carbazole groups attached at *meso*-positions (Figure 5i,j; Table 1, nos. 12–13) [45]. High photodynamic efficiency was noted in tests against *S. aureus* irradiated for 30 min (79 J/cm^2^) with white light in the wavelength range of 350–800 nm, and a high 4.5 log decrease in cell survival was observed (99.997% cell inactivation). Ballatore continued research on this conjugate (Figure 5i; Table 1, no. 12) [46] and proposed its practical use as a coating for surfaces in places particularly exposed to the development of pathogenic microorganisms.

In recent years, intensive research has been carried out on the applications of graphene quantum dots (GQDs; Figure 13). These are zero-dimensional graphene nanomaterials that exhibit the phenomenon of quantum confinement, which leads to unique physicochemical properties, such as the possibility to adjust their luminescence through controlling their formation using different synthesis methods. In addition, features such as the biocompatibility, high solubility in water, photostability, high absorption of infrared light, and ease of surface functionalization led to attempts to use quantum dots in PDT [108,109]. The properties of GQDs allow them to serve as PSs themselves, similarly to CNTs and fullerenes, instead of being only the framework or sole delivery system, such as in the case of the earlier-mentioned liposomes, polymers, sugars, VLPs, and dendrimers. Thus, their combination with porphyrins, similarly to other mentioned carbon allotropes, provides enhanced effect of PDT [108]. Further modification of their surface allows for the creation of multifunctional agents, which is also useful for synergic photothermal therapy (PTT) and for imaging and diagnostics [47]. Moreover, the GQDs can be doped with other atoms, such as nitrogen and sulfur, which results in changes in the electron density and in the GQD structure itself, possibly providing an improvement in terms of fluorescence, singlet oxygen quantum yields, and anticancer activity [109,110,111].

Managa et al. [112] created a conjugate of tetra(4-carboxyphenyl)porphyrin methyl ester (Figure 5k) with a graphene ester. This combination turned out to be stable due to the delocalized π-electrons. The compound was tested in vitro on the MCF-7 breast cancer cell line. The combination showed good biocompatibility, and the cell viability was over 90% according to the trypan blue exclusion assay. Thanks to the use of quantum dots, much better results were achieved than for porphyrin used alone when irradiated with light at a wavelength of 420 nm for 300 s. After this time, cell death was demonstrated at the level of 85%. It should be remembered that this kind of light (UV) has a limited tissue penetration rate and could be mutagenic.

There are attempts to modify porphyrins with graphene particles for the use against microbes. Yu et al. [113] presented graphene nanoribbons (GNRs) coated with cationic porphyrin (Pp4N; Figure 5l). The developed nanostructure showed a high production of ROS after irradiation with 660 and 808 nm of light. Interestingly, in the in vitro experiment, these particles showed low activity (<3 log) against a wide spectrum of Gram-positive and Gram-negative bacteria (the following strains were used: *A. baumannii*, *P. aeruginosa*, *E. coli*, *K. pneumoniae*, MRSA, *B. subtilis*, *S. epidermidis*, *Staphylococcus lentus*, and vancomycin-resistant *Enterococcus* (VRE)) [113].


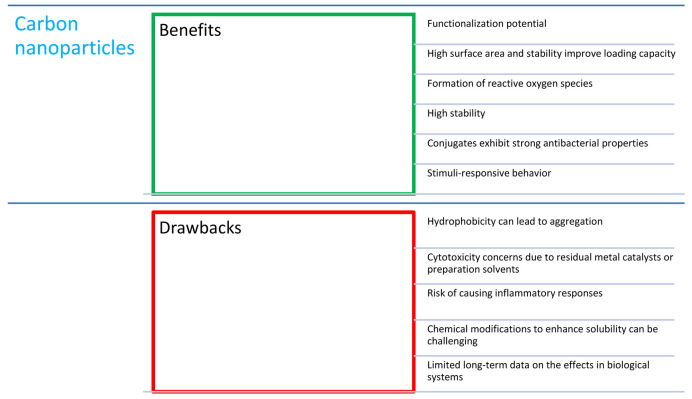


## 8. Combinations of Porphyrins with Antibodies

**Figure 15:**
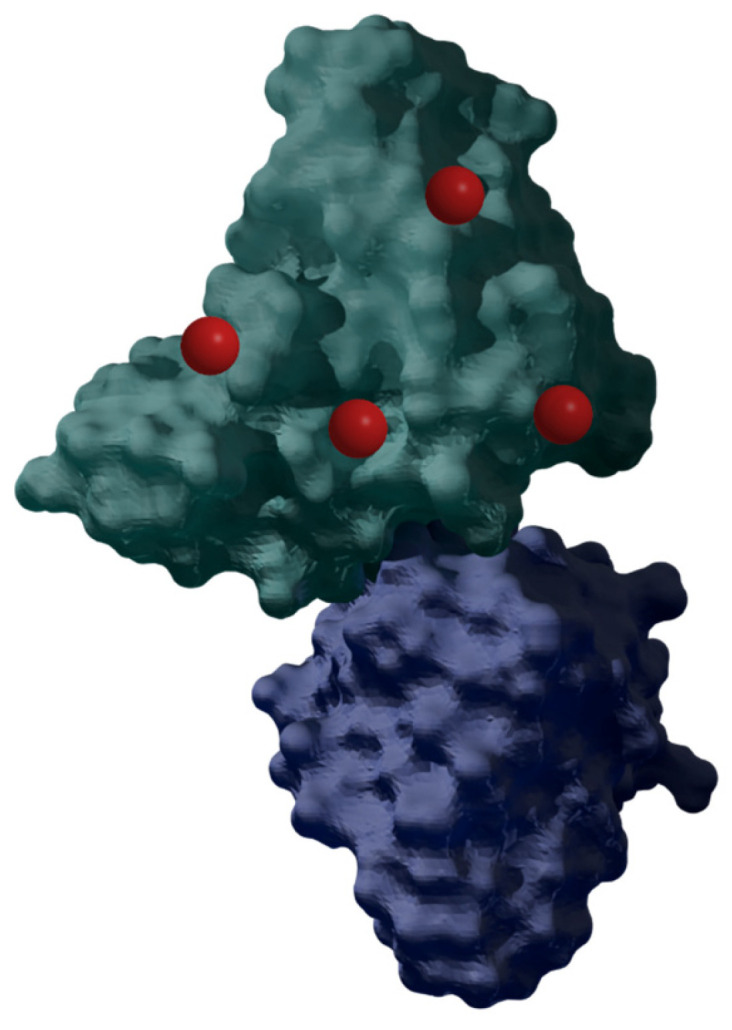
Model of the HER2 antibody doped with PS (red). Generated in Blender 4.1 based on PDB data [116,117] and modified.

Antibodies (Figure 15) were first described at the turn of the 19th and 20th centuries, but 1975 was the breakthrough year, when Milstein and Kohler discovered a method of producing monoclonal antibodies [114], for which they received the Nobel Prize in 1984. Since then, rapid progress has begun in research on their properties and potential medical applications, and, in the 1980s, antibodies were combined with porphyrins (such as hematoporphyrin; Figure 2) for the first time by Mew et al. [115]. Currently, there are many drugs approved by the FDA that are a combination of cytostatics and antibodies. They form a group of drugs called ADCs (antibody–drug conjugates) [39]. The two main antibody species studied are MAb (monoclonal antibody) and scFv (single-chain variable fragment—short variable fragments of immunoglobulins). They are often conjugated with polymer carriers to increase the solubility of the conjugate [75].

The combination of porphyrins with antibodies allows the drug to target a specific tissue thanks to specific antibody–receptor binding. This provides the possibility of treatment with mild side effects of therapy and increases the effectiveness in the combat against cancer. Additionally, the photodynamic effect induced by the porphyrin does not affect the antibody abilities; thus, the conjugation can be considered stable in the process [75,118]. An ideal porphyrin for conjugation with antibodies should contain only one functional group that would easily react with the cargo without obtaining many isomers [75].

Interestingly, Penon et al. [40] proved the effectiveness of the A_3_B-type porphyrin-AuNP-PEG conjugate (Table 1, no. 6) with the anti-erbB2 antibody (PR-AuNP-PEG-Ab) in vitro against the SK-BR-3 breast cancer cell line. The conjugate displayed an ability to generate singlet oxygen after light irradiation. The amount of singlet oxygen produced by this system resulted in a drop of approximately 14% in comparison to porphyrin alone. The cellular uptake of the PR-AuNP-PEG-Ab was confirmed by bright-field and fluorescent imaging. The effectiveness was determined by imaging the damage in the cellular structures with propidium iodide [40].


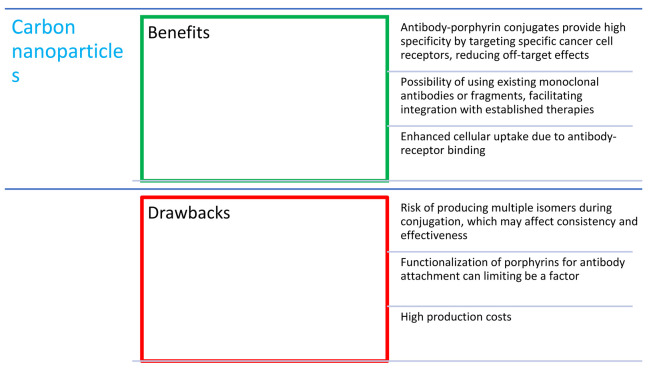


## 9. Combinations of Porphyrins with Amino Acids and Proteins

In PACT, the affinity of PSs (e.g., porphyrins) to the membrane of bacterial cells may be crucial to enable its photoinactivation [119]. Porphyrins show effective antimicrobial activity to a greater extent against Gram-positive than with Gram-negative strains, mainly due to the presence of an outer bacterial membrane with a strongly negatively charged lipid part, which can block the adhesion between the PS and bacteria [119]. O’Riordan et al. [120] illustrated the antimicrobial mechanism of action of PDT in the case of Gram-positive and Gram-negative bacterial strains. The eradication of these bacteria is based on the concept that certain PSs can accumulate on the cytoplasmic membrane, which is a critical site for causing irreversible damage to bacteria [120]. The positive charge of the PS or nanocarrier appears to promote close electrostatic interaction with the negatively charged external surfaces of the Gram-negative cell [83]. Porphyrins combined with agents that have a positive charge on their surface enhance deposition of PSs onto the outer membrane of Gram-negative bacteria and are able to improve their efficient inactivation [121].

**Figure 16:**
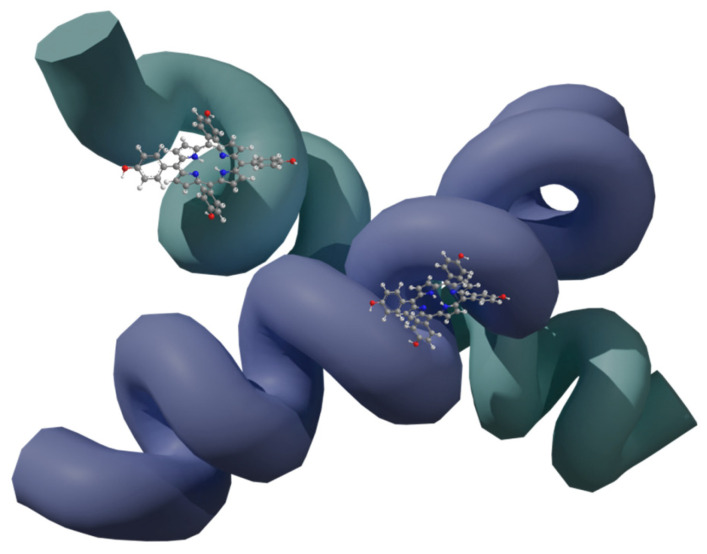
Visual representation of the magainin 2 peptide doped with PS. Generated in Blender 4.1 based on PDB data [124,125] and modified.

The combination of porphyrins with a cationic antimicrobial peptide (CAMP) is an effective method of PS delivery due to the ionic bond of the CAMP with bacterial cells [31]. These compounds also reveal a synergy of action, improving the therapeutic effect. CAMPs are a class of peptides naturally occurring in the immune system of all higher organisms, which show a high selectivity towards bacteria, leading to a non-specific immune response [122]. In a study by Dosselli et al. [42], it was proven that a neutral and hydrophobic porphyrin (Table 1, nos. 8 and 9), which was not effective against Gram-negative bacteria, was able to effectively inactivate *Escherichia coli* when conjugated with CAMPs such as buforin II and magainin 2. Buforin I is a 39-amino-acid peptide that was first isolated from the stomach tissue of the Asian toad *Bufo gargarizans*. Buforin II is its 21-amino-acid derivative, which has even stronger antibacterial activity [123]. Magainin 2 (Figure 16) is a 23-amino-acid CAMP and has potent antimicrobial activity against both Gram-positive and Gram-negative bacteria. It interrupts the bacterial membrane function by forming pores, leading to the loss of its integrity [42]. Dosselli et al. [43], continuing work on the conjugation of porphyrins with proteins (Table 1, no. 8), decided to investigate the properties of apidicins—proteins occurring naturally in the pharyngeal glands of honey bees. They have a positive charge, which ensures the accumulation on the anionic surfaces of cells containing acidic polymers, among others lipopolysaccharides or teichoic acids [44]. It was concluded that apidicins do not improve the phototoxic activity of cationic PSs, but they are able to improve the effectiveness of non-cationic PSs. Unfortunately, the combination of apidicin with high-molecular-weight porphyrins (Table 1, nos. 8–10) reduces the ability of the conjugate to penetrate the membrane of Gram-negative bacteria due to its large size. A 3 log reduction in bacterial growth to total eradication was noticed [44]. Liu et al. [119] achieved high activity in combating Gram-negative bacteria by conjugating protoporphyrin IX (Figure 2) with two sequences of lipopolysaccharide-neutralizing peptides against resistant *E. coli* and resistant *Klebsiella pneumoniae*. The monomeric and dimeric combinations were also compared, and in the latter case, higher antibacterial activity was obtained, but a still low bacterial mortality of over 99% (>2 log reduction). In a study by Xu et al. [121], a porphyrin–lysine conjugate (Figure 5m) was prepared, and its strong in vitro photodynamic antibacterial activity was proven against clinically isolated bacterial strains such as MRSA, *E. coli*, and *P. aeruginosa*. Atomic force microscopy (AFM) showed that this compound can effectively destroy the membrane and wall of bacteria, causing their disintegration, and agarose gel electrophoresis measurements revealed that DNA could also be harmed. The outcomes obtained from an in vivo study conducted on animals were very promising. The effectiveness of wound healing in rats infected with different bacteria species depended on the light dose, and the best result was recorded at a dose of 50 J/cm^2^ (650 nm), where the wound was healed within 8 days. This makes the porphyrin–lysine conjugate a promising agent of bactericidal effects both in vitro and in vivo [121].


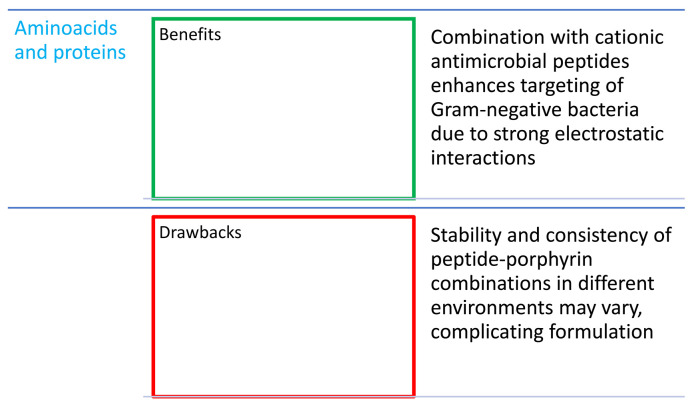


## 10. Combinations of Porphyrins with Cucurbit[7]uril

**Figure 17:**
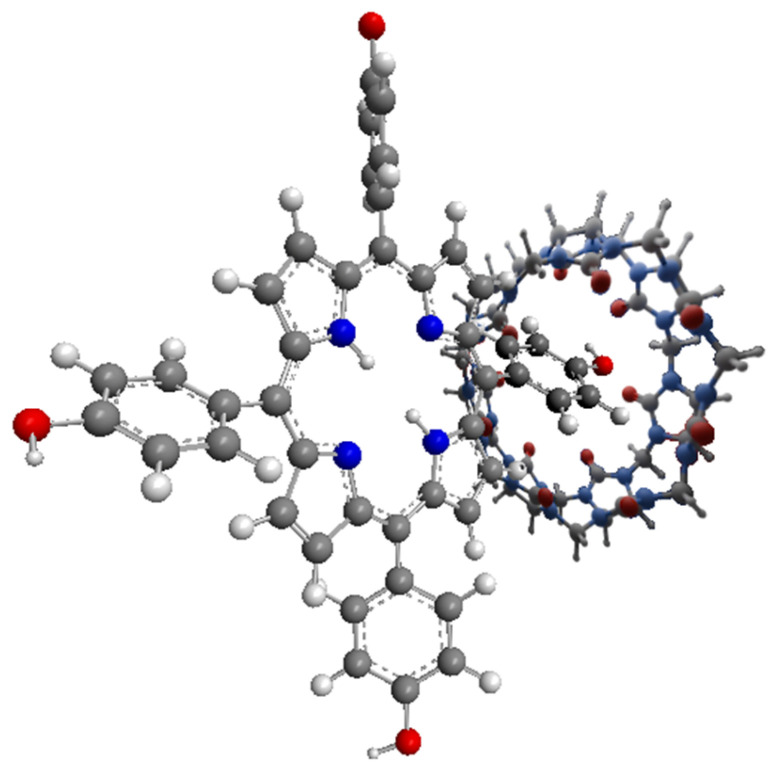
Visual representation of CB7 associated with the peripheral group of porphyrins.

Cucurbit[7]uril (CB; Figure 17) is a macrocyclic molecule composed of glycoluril monomers connected by methylene bridges [126]. Compounds from this family have become an object of interest due to such features as excellent solubility in water, the appropriate size of the cavity, which is able to fit various particles, the lack of toxicity to living cells, and the protection of encapsulated particles against external factors. This increases their chemical and thermal stability, and drug uptake in the body, and improves the overall effectiveness of the drug [126].

For the above-mentioned reasons, the researchers decided to investigate the combination of CB with porphyrins. Although cationic porphyrins (Figure 5n) have been reported to exhibit high cytotoxicity in the dark, their dark toxicity can be reduced by forming an inclusion complex with cucurbit[7]uril [127]. In this way, a complex is obtained that does not damage cells in the dark but becomes highly phototoxic when exposed to white light. Ozkan et al. [128] studied the influence of cationic porphyrin (Figure 5o) on the microorganisms. It caused a bactericidal effect in the dark on a Gram-positive bacterium (*B. subtilis*) but had no significant effect on a Gram-negative bacterium (*E. coli*). When porphyrin was combined with CB, the dark cytotoxicity decreased in both strains, whereas, after exposure to light, it increased above 3 log reduction [128].


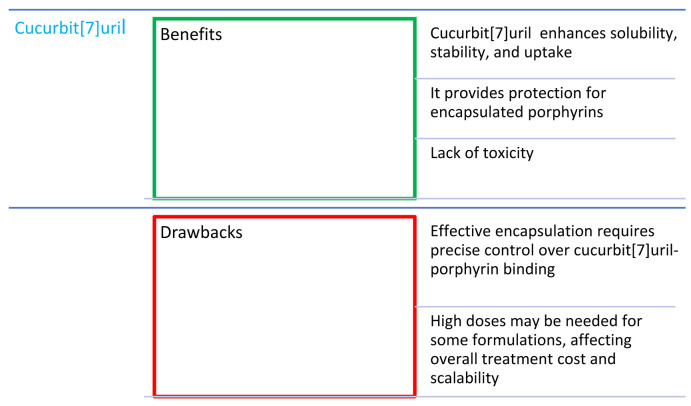


## 11. Summary

Porphyrins, despite their drawbacks, and thanks to their combination with carriers, reveal improved properties that are useful in the treatment of various types of cancer. Porphyrins, which are poorly soluble in water and prone to aggregation, thanks to the combinations with liposomes or polymers, showed their high activity. Selectivity was increased by combining them with antibodies or sugar molecules. Additionally, conjugates often gain features that are desirable for medical applications, such as reduced toxicity or the enhanced production of reactive oxygen species. The main drawback of porphyrins in PDT is their low absorption coefficient in the Q-band region, which is the cause of the high light doses needed to trigger photodynamic process. Moreover, PDT should be considered mainly as a treatment method of superficial lesions due to the low light penetration through the tissues. Thus, a new strategy is needed to trigger the photodynamic process deep inside the body. It should also be remembered that most photosensitizers also localize in many normal host tissues, e.g., the liver, spleen, and kidney, which need to be protected from irradiation.

Recently, the development of multi-resistant bacterial strains, which are a real problem in clinical practice, has become an increasingly highlighted issue. The promising solution for superficial infections, especially wounds, is PACT. Despite the limitations of the molecules themselves, combinations with various carriers make it possible to achieve effectiveness against both Gram-positive and Gram-negative strains. Additionally, modern drug formulations, like hydrogels, provide porphyrins and proper humidity to infected wounds, and cover them perfectly. This enables the reduction in healing time due to the killing of bacteria and the turning on of skin-repairing processes triggered by photodynamics, which make PACT a better solution than antibiotic therapy, especially when taking into account one of the great advantages of PSs: that bacteria do not tend to acquire resistance. An obvious limitation is the local use of porphyrins—they are unable to fight systemic infections because they require light for the initiation of the photodynamic process.

The nanomaterials described in this review, in combination with porphyrins, pave the way to the development of modern photodynamic methods.

## Figures and Tables

**Table 1:** Structures of porphyrins other than the A_4_-type presented in this work.

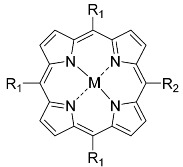 A_3_B-type porphyrins				
**No.**	**R_1_**	**R_2_**	**Metal or H_2_**	**Ref.**
1.	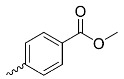	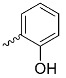	H_2_	[35]
2.	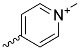	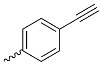	Zn	[36]
3.	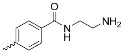	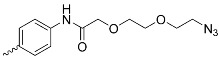	Zn	[37]
4.	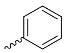	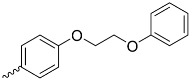	Zn, H_2_	[38]
5.	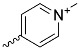	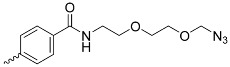	H_2_	[39]
6.	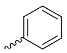	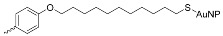	H_2_	[40]
7.	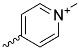	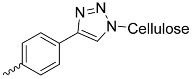	Zn	[41]
8.	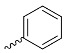	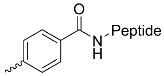	H_2_	[42,43,44]
9.	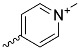	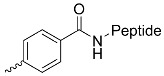	H_2_	[42,44]
10.	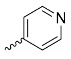	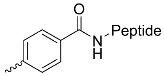	H_2_	[44]
11.	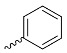	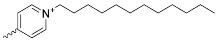	H_2_	[32]
12.	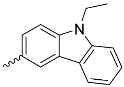	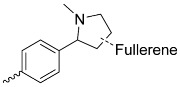	H_2_	[45]
13.	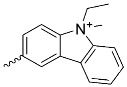	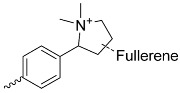	H_2_	[46]
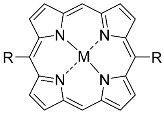 5,15-disubstituted porphyrin				
14.	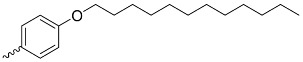		H_2_	[47]
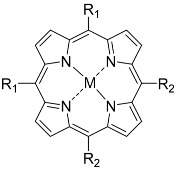 A_2_B_2_-type porphyrin				
15.	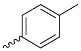	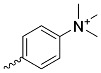	H_2_	[35]
